# Preparation, Performance Research and Field Application Practice of Temperature-Sensitive Lost Circulation Material for Shale Oil Wells

**DOI:** 10.3390/polym17172395

**Published:** 2025-09-02

**Authors:** Wenzhe Zhang, Jinsheng Sun, Feng Shen, Wei Li, Xianbin Huang, Kaihe Lv, Meichun Li, Shaofei Xue, Shiyu Wang, Hongmei Li

**Affiliations:** 1 School of Petroleum Engineering, China University of Petroleum (East China), Qingdao 266580, China; eagle.1983@163.com (W.Z.); mli@upc.edu.cn (M.L.); 2 Research Institute of Shaanxi Yanchang Petroleum (Group) Co., Ltd., Xi’an 710075, China; 3 CNPC Engineering Technology R&D Co., Ltd., Beijing 102206, China

**Keywords:** drilling fluid, lost circulation, shape memory polymer, intelligent lost circulation material, self-adaptively seal fracture

## Abstract

Drilling fluid losses into formation voids are among the major issues that lead to increases in the costs and nonproductive time of operations. Lost circulation materials have been widely used to stop or mitigate losses. In most cases, the size of the loss zone is not known, making conventional lost circulation materials unsuitable for plugging the loss zone. In this study, novel temperature-sensitive LCM (TS-LCM) particles composed of diglycidyl ether of bisphenol A (DGEBA) and 4,4′-diaminodiphenyl methane were prepared. It is a thermal-response shape-memory polymer. The molecular structure was analyzed by Fourier transform infrared spectroscopy. The glass transition temperature (*T_g_*) was tested by Different scanning calorimetry (DSC). The shape-memory properties were evaluated by a bend-recovery test instrument. The expansion and mechanical properties of particles were investigated under high temperature and high pressure. Fracture sealing testing apparatus was used to evaluate sealing performance. The mechanism of sealing fracture was discussed. Research results indicated that the *T_g_* of the TS-LCM was 70.24 °C. The shape fixation ratio was more than 99% at room temperature, and the shape recovery ratio was 100% above the *T_g_*. The particle was flaky before activation. It expanded to a cube shape, and the thickness increased when activated. The rate of particle size increase for D90 was more than 60% under 120 °C and 20 MPa. The activated TS-LCM particles had high crush strength. The expansion of the TS-LCM particles could self-adaptively bridge and seal the fracture without knowing the width. The addition of TS-LCM particles could seal the tapered slot with entrance widths of 2 mm, 3 mm and 4 mm without changing the lost circulation material formulation. The developed TS-LCM has good compatibility with local saltwater-based drilling fluid. In field tests in the Yan’an area of the Ordos Basin, 15 shale oil horizontal wells were plugged with excellent results. The equivalent circulating density of drilling fluid leakage increased by an average of 0.35 g/cm^3^, and the success rate of plugging malignant leakage increased from 32% to 82.5%. The drilling cycle was shortened by an average of 14.3%, and the effect of enhancing the pressure-bearing capacity of the well wall was significant. The prepared TS-LCM could cure fluid loss in a fractured formation efficiently. It has good prospects for promotion.

## 1. Introduction

Lost circulation is one of the most challenging problems in oil and gas drilling engineering. It is defined as the partial or complete loss of drilling fluid into formation voids or fractures during drilling [1]. It was reported that lost circulation occurs during drilling in approximately 20 to 25% of wells drilled worldwide [2]. Economic losses caused by lost circulation are estimated at USD 2-4 billion annually in lost productive time, lost drilling fluid, and materials used to cure the losses [3,4]. In severe cases, it may lead to a kick or even a blowout [5]. Chapman introduced a method to cure or prevent losses by adding granular materials to the drilling fluid in 1890 [6,7]. Since then, the drilling industry tries to solve fluid loss problems by adding lost circulation materials (LCMs) to the drilling fluids to form a plug or seal in the thief zone [8]. Conventional LCMs can be classified based on their appearance as granular, fibrous, or flaky. These materials can be used individually or in combination for better results. Non-conventional types of LCMs can be classified as cements, resins, nano-composite gels, cross linkers, and polymers; these treatments have been introduced to overcome the severe losses [9,10]. Conventional LCMs are most widely used in fields. The LCM can be effective if it bridges correctly and efficiently in the thief zone. Researchers have proposed some theories to make sure that LCMs bridge efficiently [11], such as Abram’s Rule [12], the D90 Rule [13], Vickers’s Method [14], the Halliburton Method [15], and the 3/10 and 6/5 Rule [16]. These different rules are used to optimize the particle size distribution, but they all depend on the size of the loss zone [17]. In most cases, the size of the loss zone is not known. The fracture apertures are not uniform, making the particulate approach unsuitable for plugging the fractures [18]. The particles used to plug them can either be too small and flow through or too big and may not penetrate the fractures. When curing the severe losses, the large-sized particles may plug the drilling tools. In addition, LCMs with high densities will settle in the pill preparation tank and the wellbore annulus during a lost circulation control operation. Therefore, in order to overcome the above disadvantages, a novel temperature-sensitive LCM with low density and self-adaptive sealing performance was introduced.

With the development of intellectualization and automation in drilling engineering, intelligent materials are used in the field. Shape-memory material refers to an intelligent material with an original shape; it can be deformed into a temporary shape by applying an external force and recover its original shape under external stimuli, including heat, light exposure, electrical current, or pH, etc. [19,20]. Shape-memory polymers (SMPs) offer a variety of chemical structures, reduced manufacturing costs, ease of preprocessing, lightweight properties, high restorative deformation, and adjusted recovery temperatures [21,22,23]. Thermal-response SMPs are one of the most extensively studied systems, and their shape memory is generally based on the glass transition temperatures (*T_g_*) [24,25]. When heated above the *T_g_* of the SMP, the material can be deformed to a temporary shape by applying an external force, and then, when cooled to below the *T_g_*, the segmental motion of the polymer chain is frozen, and it can maintain the temporary shape. When heated above the *T_g_*, the SMP can release the stored entropy, and the SMP can recover to its original shape [26,27].

Based on the shape-memory properties of the thermal-response SMPs, the material can be deformed into a small-sized particle and therefore transported through the drilling tools, penetrating the fractures. Then, it can expand above its *T_g_* within the fracture and self-adaptively seal the fracture [28,29]. In this study, a novel temperature-sensitive LCM that is made out of thermal-response SMPs is introduced. Firstly, shape-memory epoxy resin (SMEP) was prepared, and the sample was in the form of the cuboid. Then the SMEP samples were compressed into a flaky shape by a hot-pressing machine above its glass transition temperature. Then, they cooled down to room temperature, and the load was removed. Finally, it was crushed into particles by a pulverizer. The LCM particles are of a small size so that they can go through the drilling tools without plugging them. Then they will be activated to expand at the formation temperature within the fracture. The expansion of the particles could bridge and seal the fracture without the width of the fracture being known. The thermal and mechanical properties, shape-memory behavior, expansion properties, and sealing properties of the temperature-sensitive LCM were investigated.

## 2. Experiment

### 2.1. Materials

Diglycidyl ether of bisphenol A (DGEBA) was purchased from the Shanghai Coking & Chemical Development Company, Shanghai, China (the epoxy equivalent was 0.51). 4,4′-diaminodiphenyl methane (DDM), as a curing agent, was sourced from Shanghai Yi’en Chemical Technology Co., Ltd. (Shanghai, China).

### 2.2. Preparation of Temperature-Sensitive LCM

**Figure 1:**
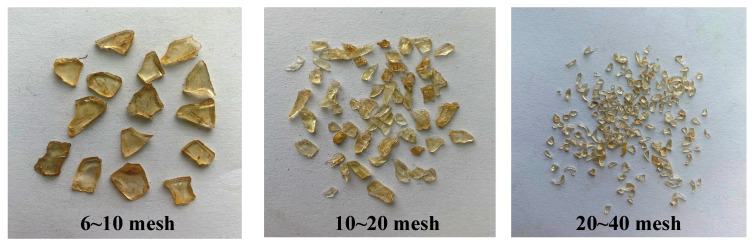
Temperature-sensitive LCM particles with different sizes.

The reaction was carried out in a 50 mL beaker equipped with a mechanical stirrer and heating device. DGEBA (50 g) was added into the beaker at 60 °C under stirring for 10 min. Then the DDM (8.42 g) was dropped into the beaker under stirring to form a homogeneous solution for 20 min. The mixture was degassed at 60 °C under a vacuum to remove trapped air and then poured directly into a Teflon mold. The synthesis involved placing the mixture in a vacuum-drying oven for 30 min of degassing. The mold was pre-coated with Vaseline to facilitate demolding and preheated in an oven. The mixed raw materials were then poured into the mold and thermally cured to form the shape-memory polymer. The mold was a cuboid, and the length, width, and height were 100 mm, 10 mm, and 5 mm, respectively. It was thermally cured in an air convection oven at 80 °C for 2 h and then at 130 °C for 2 h. Then the casting was removed from the mold. The casting was compressed at 15 MPa for 15 min by a hot-pressing machine at 100 °C. The SMP had a flaky shape after pressing. Then, it cooled down to room temperature, and the load was removed. Finally, the compressed castings were crushed into flaky particles by a pulverizer. Temperature-sensitive LCMs with different particle sizes were obtained (Figure 1).

### 2.3. Fourier Transform Infrared Spectroscopy Test

The chemical structure of the prepared LCM was analyzed by Fourier transform infrared spectroscopy (Bruker Corporation, Bremen, Germany, Vertex 70 FT-IR device) in the range of 400–4000 cm^−1^ using the attenuated total reflection (ATR) method.

### 2.4. Thermal Analysis

Differential scanning calorimetry (DSC) testing was carried out on a TA Q20 instrument (TA Instrument, New Castle, DE, USA), using nitrogen as the purged gas, at a scan rate of 4 °C/min, with temperature ranging between 20 and 300 °C.

### 2.5. Shape-Memory Test

**Figure 2:**
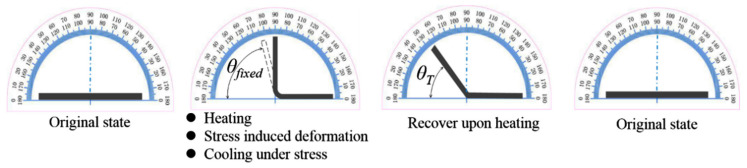
Bend-recovery shape-memory test instrument.

The cuboid casting specimen had dimensions of 100 mm × 10 mm × 5 mm. The shape-memory test was performed using the following steps and a bend-recovery shape-memory test instrument [30]: (1) The cuboid casting is placed into an oil bath and held for 10 min at *T_g_* + 20 °C. (2) The casting folds into a 90° rectangular shape, as shown in Figure 2. (3) The folded casting is placed into cold water for 10 min and then taken out. (4) The folded casting is kept at room temperature for 30 days, and then the bending angle is measured (*θ_fixed_*). (5) The folded casting is heated again from 20 °C at a rate of 1 °C/min. The folded casting begins to recover its original state above *T_g_*. The recovery angle is *θ_T_* at the temperature *T*. The shape fixation ratio (*R_f_*) and shape recovery ratio (*R_r_*) are calculated using the equations *R_f_* = *θ_fixed_*/90° × 100% and *R_r_* = (*θ_fixed_* − *θ_T_*)/*θ_fixed_* × 100%.

### 2.6. Expansion Test

The temperature-sensitive LCM particles need to expand to their original state at the bottomhole under high temperature and high pressure. The expansion property was tested using a high-temperature and high-pressure airtight apparatus. The temperature-sensitive LCM particles were mixed with a water-based drilling fluid and poured into the cell. Then the top cover was screwed tightly in order to build up pressure in the cell [6,11]. Then, the cell was heated to 120 °C and the pressure was raised to 20 MPa by an oil hydraulic pump. The test was run for 30 min. Dry sieve analysis tests were conducted to measure D50 and D90 of the particles before and after activation. The rates of increase in particle size of D50 and D90 were calculated.

### 2.7. Mechanical Test

The plugging zone formed by LCMs inside the fracture will bear different stresses, including fracture closure stress, wellbore fluid pressure, and formation pressure [31,32,33]. The LCM bridge is expected to have good crush strength or crush resistance for it to be durable [34]. Dry sieve analysis tests were conducted to measure D10, D50, and D90 of the particles before crushing. A sample holder was used to store the LCM particles. A press was used to simulate the fracture closure stress on the LCM particles. Then a pressure of 20 MPa was applied for 30 min. After crushing, particles were again sieved to estimate D10, D50, and D90. The size degradation rate of particles was used to quantify the LCM size change and is described by Equation (1). The heating jacket wrapped around the sample holder to simulate the activation temperature.

(1) $$SC=\left(\frac{D\left(10/50/90\right)-D^{\prime }\left(10/50/90\right)}{D\left(10/50/90\right)}\right)\times 100\%$$

where *SC* is the size degradation rate of particles. *D* (10/50/90) is the value of the particle diameter at 10%, 50%, and 90% in the cumulative size distribution before an applied load. *D*′ (10/50/90) is D10, D50, and D90 after particle size degradation.

### 2.8. Sealing Fracture Test

**Figure 3:**
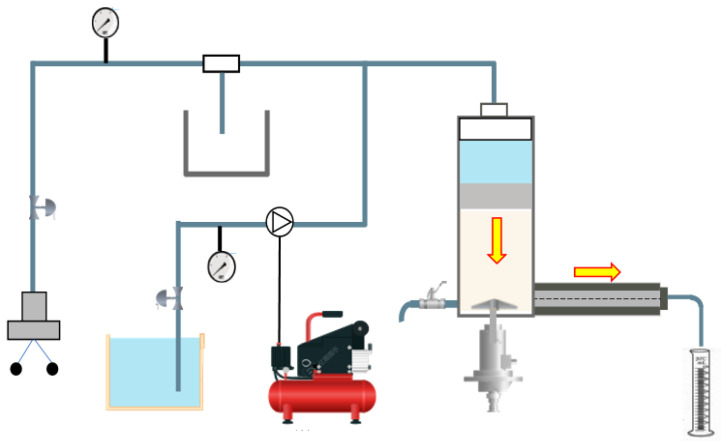
Schematic of the LCM testing apparatus with tapered fracture slot.

**Figure 4:**
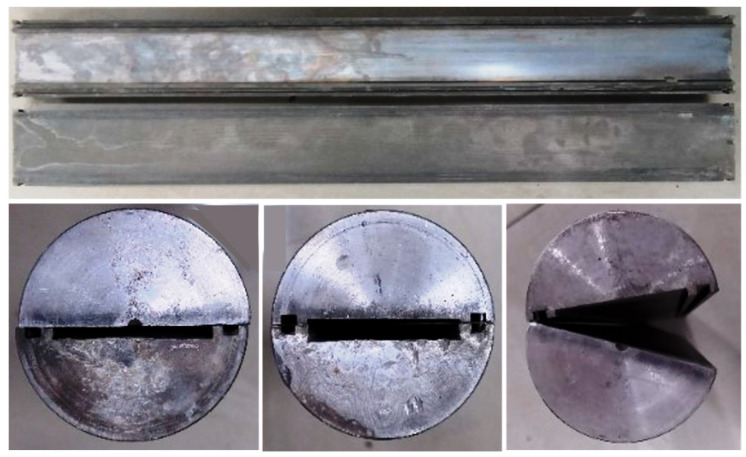
Tapered slots with various fracture sizes for fracture sealing testing.

In order to evaluate the properties of fracture sealing of LCM particles, a fracture sealing testing apparatus (Figure 3) with tapered and slotted stainless steel disks (Figure 4) that simulate natural fractures was adopted. Yellow arrows indicate the fluid’s way through the system. Nitrogen was injected to pressurize the piston to push the drilling fluid containing LCM treatments through the tapered slot. Tapered slot specifications are shown in Table 1. The sealing apparatus could withstand up to 8 MPa and 180 °C. Firstly, tapered slots were placed inside the core gripper. Drilling fluid containing LCMs (1000 mL) was introduced into the test cell and heated to the desired temperature. Then the rotor was turned at a rate of 100 rpm. The pressurization system could provide a continuous flow of nitrogen to push the drilling fluid containing LCMs through the long, tapered slot. If total loss occurred during pressurizing, the plugging failed. If fluid loss stopped during pressurizing, the pressure was kept constant at 1.0 MPa and kept stable for 10 min. If there was no fluid loss within 10 min, then this step was repeated, pressurizing to 1.0 MPa and holding for 10 min. The pressure at which the plugging zone broke and fluid loss was observed from the outlet was noted. This pressure was noted as the breaking pressure. Because the sealing apparatus could withstand up to 8 MPa, the test was also stopped when the applied pressure was 8.0 MPa due to test limitations.

## 3. Results and Discussion

### 3.1. Fourier Transform Infrared Spectroscopy

**Figure 5:**
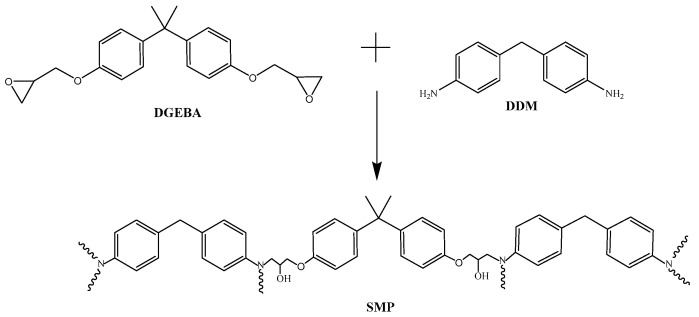
Chemical reaction mechanism for SMP.

**Figure 6:**
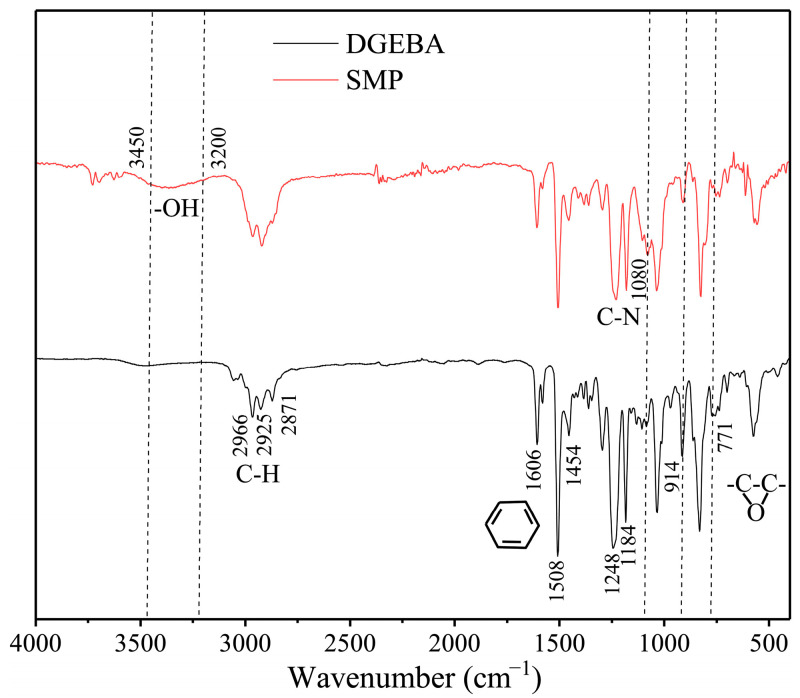
FT-IR spectra of epoxy resin and prepared shape-memory polymer.

The chemical reaction mechanism for the SMP is depicted in Figure 5. The FT-IR spectra of DGEBA and the SMP are shown in Figure 6. C-H stretching vibration absorption appeared in the range from 2860 to 3000 cm^−1^. The bands at 1454, 1508, and 1606 cm^−1^ are characteristic absorption peaks of benzene. There were no changes in these bands, and due to the benzene ring in DGEBA, no reaction occurred with the curing agent. The bands at 1184 and 1248 cm^−1^ are C–O–C stretching vibrations in DGEBA. The decrease in peak intensities of the bands at 914 and 771 cm^−1^ (epoxy stretching vibration) in the SMP was due to the reaction of the epoxide group upon the addition of DDM. The epoxide group reacted with the hydrogen of the amine group to form the hydroxyl group. The characteristic peak of -OH increased (3200~3450 cm^−1^). The band in the SMP at 1080 was the C–N stretching vibration. The appearance of C–N and –OH suggests that the epoxy resin and curing agent were successfully involved in the reaction.

### 3.2. Thermal Property

**Figure 7:**
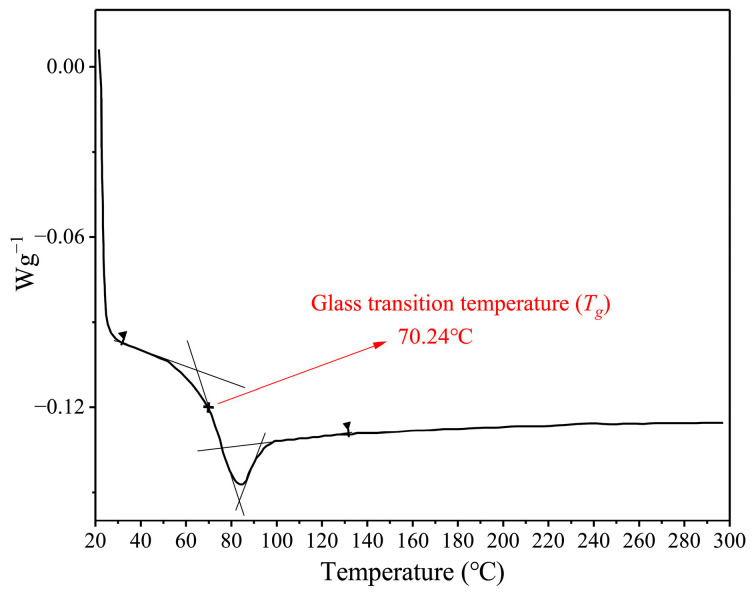
DSC curves of prepared shape-memory polymer.

The thermal property, specifically the glass transition temperature (*T_g_*), of the SMP was confirmed by DSC. The DSC heating curves of the SMP are shown in Figure 7. The glass transition of the SMP is characterized by a shift in the baseline. The glass transition temperature is determined by the midpoint of the tangent line and baseline. The glass transition temperature of the SMP is 70.24 °C.

### 3.3. Shape-Memory Properties

Shape-fixing performance reflects the ability of SMPs to retain a temporary form at room temperature for a period of time. A high shape fixation ratio contributes to storing at room temperature, and the SMP will not recover the original shape at low temperatures. The *R_f_* of the SMP is shown in Table 2. It is evident that the SMP has a high shape fixation ratio at different temperatures (20, 25, 30, and 35 °C). The SMP has good shape-fixing performance. The small LCM particles have difficulty to recover the original shape at room temperature.

**Figure 8:**
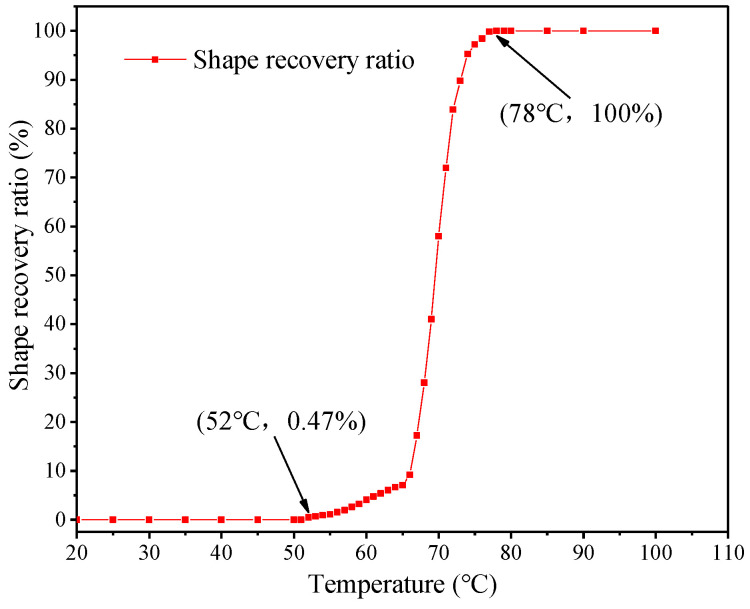
Shape recovery ratio of prepared shape-memory polymer vs. temperature.

Shape recovery performance reflects the ability of SMPs to recover their original shape after thermo-mechanical deformation at high temperatures. The *R_r_* of the SMP is shown in Figure 8. The SMP began shape recovery when the temperature was 52 °C. When the SMP is heated to 78 °C, an *R_r_* of over 99% means recovering the original shape. The small LCM particles will be activated to expand and recover their original shape fully at the formation temperature. Our results show that high *R_f_* and *R_r_* yield good shape-memory lost circulation materials. The time required to achieve 100% shape recovery was 20 min at 60 °C, 8 min at 70 °C, and 2 min at 80 °C.

### 3.4. Expansion Properties

**Figure 9:**
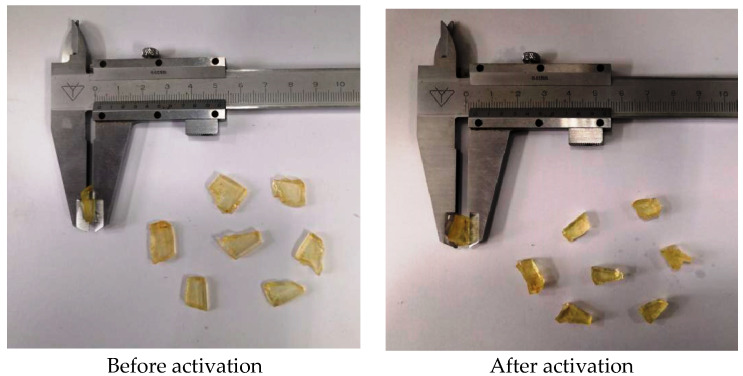
Photo of temperature-sensitive LCM particles before and after activation.

The LCM particles have an irregular flaky shape before activation and expansion. The average thickness is 1 mm. After activation and expansion, the average thickness increases to 5 mm. Figure 9 shows the LCM particles before and after activation.

**Figure 10:**
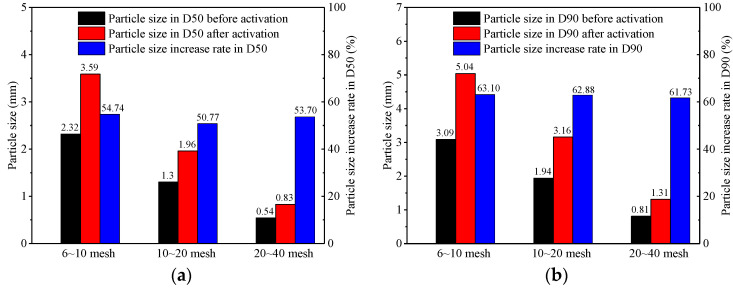
Particle size and percentage change in LCM particles before and after activation under high temperature and high pressure. (**a**) Particle size and percentage change in D50. (**b**) Particle size and percentage change in D90.

Figure 10 shows the particle size and size increase rate in D50 and D90 of LCM particles before and after activation under high temperature and high pressure. It can be seen that the particle size increases after expansion. The rate of particle size increase in D50 is more than 50%, and the rate of particle size increase in D90 is more than 60%. The expansion of the LCM particles could bridge and seal the fracture under high temperature and high pressure.

We conducted comparative tests on rheological properties and fluid loss performance before and after adding the material. The base drilling fluid exhibited an AV of 30 mPa·s, PV of 20 mPa·s, and API fluid loss of 3.0 mL. After adding 8–20 mesh TS-LCM particles and aging at 120 °C for 16 h, the drilling fluid maintained an AV of 31 mPa·s, PV of 20 mPa·s, and API fluid loss of 2.8 mL. The negligible changes demonstrate minimal impact on rheological and filtration properties. This compatibility is attributed to the material’s inert nature—it absorbs neither water nor oil and only expands in response to temperature stimuli.

### 3.5. Mechanical Properties

**Figure 11:**
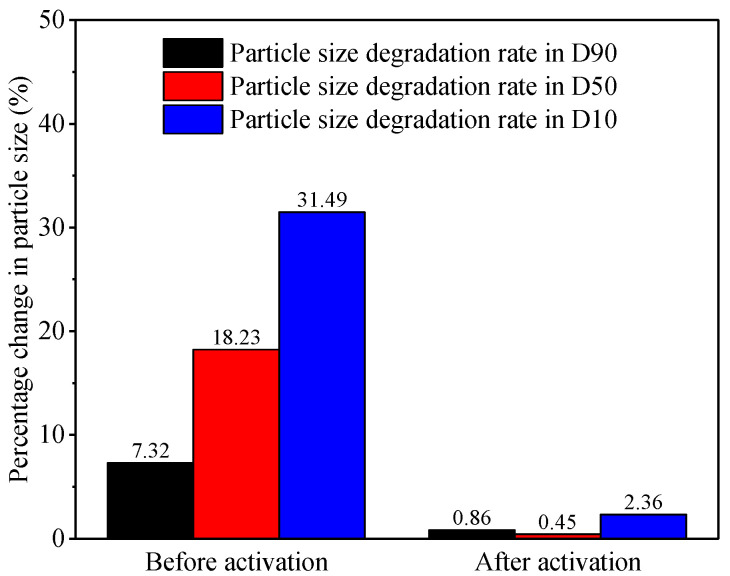
Size degradation rate of LCM particles before and after activation.

Figure 11 shows the crush test results of LCM particles under room temperature and activation temperature. The percentage change in D10, D50, and D90 is high under room temperature. The LCM particles are referred to as a brittle material, and the particle size decreases when crushed at room temperature. The percentage change in D10, D50, and D90 decrease obviously under the activation temperature. The activated LCM particles have high crush strength, and it could prevent the compressive-crush instability of the formed seal.

At room temperature, the SMP is typically a rigid plastic. When heated to the transition temperature (*T_g_*) of the shape memory, the molecular chains move fast and the shape-memory polymer becomes an elastomer [22,23]. Conventional lost circulation materials such as calcium carbonate exhibit degradation rates of 15% for D90, 37.2% for D50, and 86.32% for D10. In contrast, TS-LCM particles demonstrate superior crush resistance due to their significantly lower degradation rates. The LCM particles transform from brittle material to ductile material under the activation temperature. Molecular chains release entropic energy, thus driving the particles to recover their original shape while unloading the pressure under the high temperature.

### 3.6. Sealing Properties

Addition of LCMs to carrier drilling fluids is the most frequently used method for lost circulation control [35,36]. The drilling fluid must have good carrying capacity to prevent LCM settling in the pill preparation tank and in the wellbore annulus [37]. In the field, bentonite mud with viscosifiers is found to be effective, with an improved fluid-carrying capacity. To overcome the problem, High-Viscosity Sodium Carboxymethyl Cellulose (CMC-HV) was added to 6% by weight bentonite mud. The formulations and rheology of carrier drilling fluid are illustrated in Table 3.

The fracture sealing testing apparatus was used to test the sealing efficiency of the temperature-sensitive LCM. The research indicates that combinations of LCMs improve sealing performance more than using just one type of LCM [38]. Most commonly used granular and fibrous LCMs in the field were selected to evaluate sealing efficiency, including sized calcium carbonate (SCC) and polypropylene fiber (PPF). Table 4 shows LCM formulas for lost circulation control of water-based drilling fluids.

Table 5 shows the test results of LCM treatments in a 2 × 1 mm width tapered slot. Experiments were performed at 25 °C and 90 °C, respectively. At 25 °C, there were no bridge particles in case 1# and 2# for the 2 × 1 mm width tapered slot, so total loss occurred. At 90 °C, for the control sample in case 1#, there was no sealing pressure, and total loss occurred because of the lack of bridge particles, while for case 2#, addition of TS-LCM promoted high sealing pressure (8 MPa) and low fluid loss (20 mL). The TS-LCM particles were activated at 90 °C, and the particle size increased after expansion. Then the expandable TS-LCM particles bridged the open area within the fracture, and fine SCC particles and PPF filled the void spaces between the coarse TS-LCM particles, maintaining higher seal integrities.

**Figure 12:**
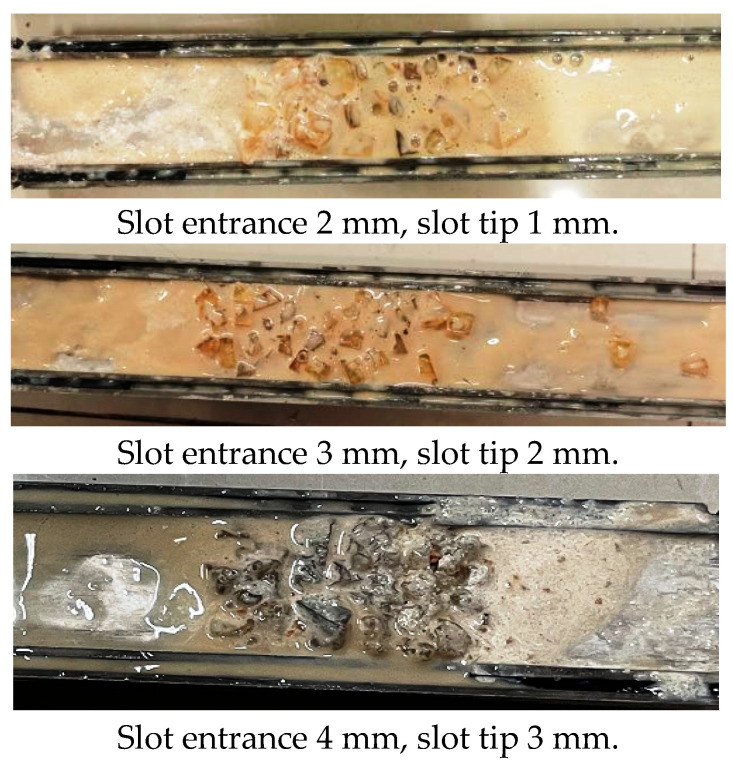
LCM distribution in the slots after the sealing test.

In most cases, the size of the formation fracture is not known. In order to understand the self-adaptive sealing performance of the temperature-sensitive LCM particles, three tests were performed to evaluate the sealing performance in fractures with different entrance widths. Table 6 shows the test results of case 2# in sealing tapered slots with different entrance widths. At 90 °C, temperature-sensitive LCM particles were activated, and the particle size increased after expansion. Expandable particles can bridge within fractures with different entrance widths. The LCM formula in case 2# could seal the tapered slot with entrance widths of 2 mm, 3 mm, and 4 mm without changing the LCM formulation. There is high sealing pressure and low fluid loss for every slot. We opened the fracture slot after the test and, as shown in Figure 12, the temperature-sensitive LCM particles, sized calcium carbonate, and polypropylene fiber evenly distributed to synergistically form a seal within the fracture for every slot (red dashed frame). After the expandable TS-LCM particles’ bridging, fine-sized calcium carbonate particles and polypropylene fiber could fill the formed voids, which was favorable for forming a tighter seal and reducing fluid loss. The expansion of the LCM particles could bridge and seal the fracture without the width being known.

## 4. Mechanism of Sealing Fracture

A thermally induced shape-memory cycle of the temperature-sensitive LCM particles is shown. The molecular chains of the prepared SMP are stable below *T_g_*, so it is in a rigid plastic state. The temperature-sensitive LCM particles are cube-shaped and thick. The molecular chains are in a high-mobility state, and the SMP is activated to be an elastomer when heating above *T_g_*. The molecular chains are flexible in this stage. The temperature is kept above *T_g_,* and an external compression load is applied by a hot-pressing machine. The macroscopic shape changes from cube to flake. Then the SMP is cooled down below *T_g_* while the external compression load is maintained. The molecular chains are freezing and in a low-mobility state below *T_g_*. So the SMP has a fixed, flaky shape even though the external load is removed. The flaky materials are the temperature-sensitive LCM particle products. Reheating above *T_g_*, the molecular chains are in a high-mobility state, returning to their highest entropy state, and the flaky temperature-sensitive LCM particles will recover to the cube shape.

The sealing performance of different LCMs of various shapes in the fractures was studied [39]. The flaky LCMs were strongly adaptable to variation in the fracture opening. When the size of the flaky LCMs is larger than the fracture opening, they can enter the fracture horizontally. The advantages of the cube LCMs are their rapid plugging and high plugging efficiency. When the particle size matches the fracture width, they will bridge and seal the fracture quickly. However, the plugging quality of the cube LCMs was affected by the particle size. If the particle size of the cube LCMs was larger than the fracture opening, the particles bridged and filled at the entrance of the fracture, and there was no sealed barrier inside the fracture. So the cube LCMs were poorly adaptable to variation in the fracture opening. The temperature-sensitive LCM particles have a flaky shape at room temperature. They are carried by drilling fluid and can easily enter the formation fracture without a known width of the fracture. Then they will be activated to recover a cube shape at high formation temperatures. The expansion of the particles to a cube shape could bridge and seal the fracture rapidly. The prepared temperature-sensitive LCM particles could cure fluid loss in fracture formations efficiently.

## 5. Field Test

Yan’an is located in the Ordos Basin, which has rich terrestrial shale oil and gas geological resources and huge development potential. More than 80% of the newly proven geological reserves in the region are tight oil reservoirs and shale oil reservoirs, and shale oil will become the main replacement resource for stable production in the future. Since 2011, during the exploration of shale oil resources in the Changchang 7 shale oil resource in the southwestern part of the Ordos Basin, a large number of wells have found rich oil and gas indicators in black organic shale, and the low oil flow obtained by fracturing a few wells proves the existence of shale oil resources in organic shale. The 15 horizontal wells that were initially drilled had certain technical problems using local water-based drilling fluids, and 5 horizontal wells were completed ahead of schedule because the problem of lost return leakage could not be solved [40].

The depth of loss zones in field applications ranges approximately from 2000 to 3000 m, with temperatures between 60 °C and 80 °C. Therefore, a *T_g_* of 70.24 °C ensures optimal shape recovery, precisely within the target loss zones. During operations, drilling is typically paused for pressure-bearing plugging operations. When the lost circulation material enters the loss zone, it gradually restores its original shape, triggered by the elevated downhole temperature, thereby achieving effective bridging and sealing.

In order to solve the problem of low pressure-bearing capacity and formation leakage caused by the development of bedding cracks in mudstone and shale formations, the developed LCM and local saltwater polymer drilling fluid system achieved good compatibility, successfully completed the field construction of 15 shale oil horizontal wells in the Yan’an area, increased the equivalent circulating density of drilling fluid leakage by an average of 0.35 g/cm^3^, raised the success rate of plugging malignant leakage from 32% to 82.5%, and shortened the drilling cycle by an average of 14.3%. It can be seen that the developed LCM can adaptively fill the micro-cracks around the well, and the effect of strengthening the pressure-bearing capacity of the well wall is significant.

Based on our comprehensive technical research results, a well-by-well strategy has been established to establish a leak prevention and plugging process technology plan of “advanced diagnosis, accurate plan, and real-time processing” [41], which has been fully promoted on site.

## 6. Conclusions

In this study, temperature-sensitive LCM particles made out of thermal-responsive shape-memory polymers were prepared. The activation temperature was 70.24 °C. The shape fixation ratio was more than 99% at room temperature, so it was difficult to recover the original shape with a small size at low temperatures. The particles were flaky before activation so they could go through the drilling tools without plugging them. The shape recovery ratio was 100% when heated to 78 °C, which meant that the small TS-LCM particles would be activated and expand to a cube shape fully, and the thickness increased. The increase rates of particle size in D50 and D90 were more than 50% and 60%, respectively. The activated LCM particles had high crush strength, and it could prevent the compressive-crush instability of the formed seal. More importantly, the expansion of the particles to a cube shape could bridge and seal the fracture rapidly. It could self-adaptively bridge and seal the fracture without the width being known. At 90 °C, when used in combination with other conventional LCMs, the addition of TS-LCM particles could seal the tapered slot with entrance widths of 2 mm, 3 mm, and 4 mm without changing the LCM formulation. There was high sealing pressure (8 MPa) and low fluid loss for every slot. After field practice in shale oil wells, the prepared TS-LCM could cure fluid loss in fractured formations efficiently. However, the shape-memory polymer developed in this study has a relatively singular temperature response. Future work will focus on developing lost circulation materials adaptable to formations with different temperature gradients, thereby broadening the applicable temperature range.

## Figures and Tables

**Table 1:** Tapered slot specifications.

Slot Code	Diameter (mm)	Length (mm)	Slot Entrance (mm)	Slot Tip (mm)
1	38	90	2	1
2	38	90	3	2
3	38	90	4	3

**Table 2:** Shape fixation ratio of prepared SMP.

Temperature (°C)	Fixed Angle (°)	Angle After 30 Days, *θ_fixed_* (°)	Shape Fixation Ratio, *R_f_* (%)
20	90	89.8	99.78
25	90	89.8	99.78
30	90	89.5	99.44
35	90	89.5	99.44

**Table 3:** Formulations and rheology of carrier drilling fluid.

Components	Carrier Drilling Fluid
Bentonite (wt %)	6.0
CMC-HV (wt %)	0.4
Apparent viscosity (mPa·s)	52.0

**Table 4:** LCM formulas for lost circulation control of water-based drilling fluids.

Testing Formula	SCC (20~40 Mesh)	SCC (40~80 Mesh)	PPF	TS-LCM (20~40 Mesh)	TS-LCM (40~80 Mesh)
Case 1#	2.0%	3.0%	0.2%	-	-
Case 2#	2.0%	3.0%	0.2%	3.0%	2.0%

**Table 5:** Test results of LCM treatments in sealing a 2 × 1 mm width tapered slot.

Temperature (°C)	Testing Formula	Sealing Pressure (MPa)	Fluid Loss (mL)
25	Case 1#	0	Total loss
	Case 2#	0	Total loss
90	Case 1#	0	Total loss
	Case 2#	8	20

**Table 6:** Test results of case 2# in sealing tapered slots with different entrance width.

Testing Formula	Temperature (°C)	Slot Entrance (mm)	Slot Tip (mm)	Sealing Pressure (MPa)	Fluid Loss (mL)
Case 2#	90	2	1	8	20
		3	2	8	35
		4	3	8	70

## Data Availability

Data available in a publicly accessible repository in FigShare at https://doi.org/10.1021/acs.jmedchem.5b00788.
